# Laparoscopic retrieval of an intra-abdominal sewing needle following unrecognized penetration: a case report

**DOI:** 10.1093/jscr/rjag1012

**Published:** 2026-10-31

**Authors:** Linda M Beukes, Jade Watson, Ahmed Faidh Ramzee Abdul Aziz

**Affiliations:** Department of General Surgery, John Hunter Hospital, Lookout Road, New Lambton Heights, NSW 2305, Australia; Department of General Surgery, John Hunter Hospital, Lookout Road, New Lambton Heights, NSW 2305, Australia; Department of General Surgery, John Hunter Hospital, Lookout Road, New Lambton Heights, NSW 2305, Australia

**Keywords:** foreign body, sewing needle, laparoscopy, intraoperative radiography, transcutaneous penetration

## Abstract

Unrecognized transcutaneous penetration by a small sharp foreign body is rare and may present without a clear history of injury. A 50-year-old woman presented with abdominal pain and was found to have a 4-cm linear metallic foreign body within the peritoneal cavity. After initial observation, she represented with recurrent pain and radiographic migration of the object, later recalling that she may have rolled onto a sewing needle while asleep. At diagnostic laparoscopy the needle was not immediately visible. Sequential intraoperative radiographs, using a laparoscopic grasper as a radiopaque reference point, enabled localization of the needle within greater omentum adjacent to the transverse colon. It was removed laparoscopically without visceral injury. This case demonstrates the value of targeted intraoperative radiography when a small radiopaque foreign body cannot be identified during minimally invasive exploration.

## Introduction

Sharp intra-abdominal foreign bodies may enter through ingestion, iatrogenic introduction, or penetrating trauma. Unrecognized transcutaneous entry is uncommon and may delay diagnosis. Sharp objects can migrate and injure bowel, mesentery, or vessels, making accurate localization and timely management important. We report laparoscopic retrieval of a sewing needle after unrecognized transcutaneous penetration, using intraoperative radiography when direct visualization was unsuccessful.

## Case report

A 50-year-old woman was referred to the emergency department with a 1-day history of acute central and epigastric abdominal pain. She denied bowel or urinary symptoms. Examination demonstrated mild central abdominal tenderness without guarding or peritonism and sinus tachycardia of 130 beats/min. Laboratory investigations were unremarkable. Computed tomography demonstrated an approximately 4-cm linear extraluminal metallic foreign body in the left abdomen, without free gas, collection, or surrounding inflammatory change.

She was admitted overnight for observation but could not initially identify a possible mechanism of entry. Her pain resolved and repeat plain radiography showed the object in a similar position. As its relationship to her symptoms remained uncertain, she was discharged with close surgical follow-up and return precautions.

The following day she represented with recurrent abdominal pain and left lower quadrant tenderness. Repeat radiography demonstrated interval inferior migration of the foreign body without free subdiaphragmatic gas. She then recalled falling asleep while sewing and believed she may have rolled onto a sewing needle. Given recurrent symptoms, demonstrated migration, and the risk of visceral or vascular injury, operative retrieval was undertaken.

Diagnostic laparoscopy identified a small haematoma at the root of the small-bowel mesentery, without active bleeding or enteric contamination. The small bowel, colon, and visualized solid organs were otherwise intact. The needle was not visible despite careful inspection. Sequential intraoperative radiographs were therefore obtained from different projections. A fenestrated laparoscopic grasper was used as a radiopaque reference point, allowing triangulation of the needle (Fig. 1).

**Figure 1:**
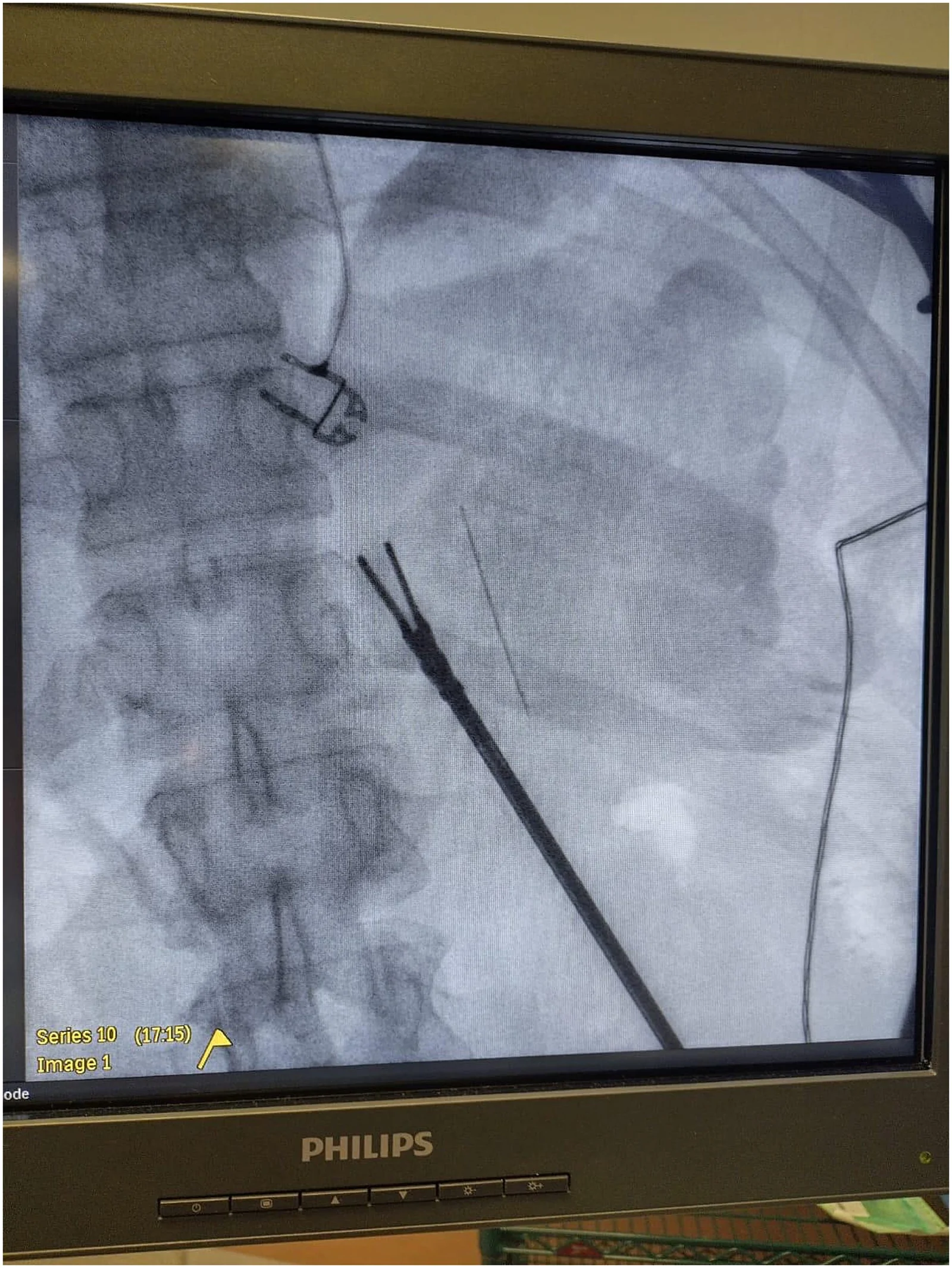
Image of localization of needle intra-operatively using X-ray and a fenestrated grasper.

The needle was encapsulated within the greater omentum adjacent to the transverse colon. A small opening was created with diathermy and the needle was removed atraumatically through a 5-mm port (Supplementary Video S1). Systematic inspection showed no bowel perforation, serosal injury, or ongoing haemorrhage, and the procedure was completed laparoscopically. The patient was discharged on postoperative day 1. She subsequently represented with mild periumbilical cellulitis without a collection, which resolved with oral antibiotics; no further complication was documented.

## Discussion

Intra-abdominal foreign bodies arise through accidental penetration, ingestion, iatrogenic introduction and, less commonly, deliberate self-harm, or interpersonal violence. Deliberate transabdominal insertion has been described in psychiatric and custodial populations, but the rarity of this mechanism means reliable population incidence estimates are not available [1]. Sharp retained objects are clinically important because migration can cause perforation, abscess, fistula, and vascular or mesenteric injury [2, 3]. The greater omentum may encapsulate foreign material and limit further migration, as occurred in this case (Fig. 2) [4].

**Figure 2:**
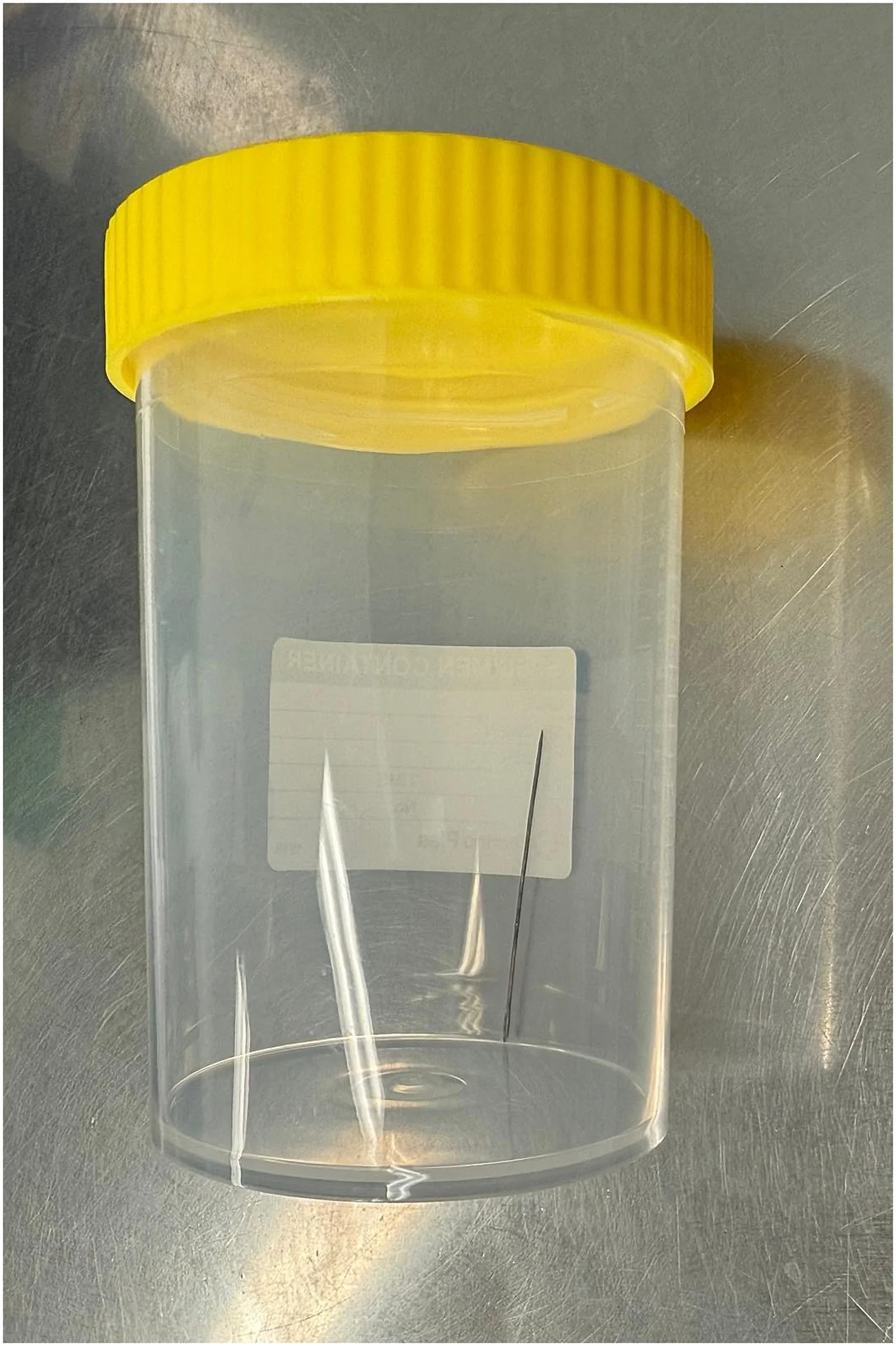
Image of retrieved needle in a specimen jar.

Management should be individualized according to the object’s composition, shape, location, mobility, and associated injury. A mobile sharp intraperitoneal object generally favours removal because of the risk of subsequent visceral injury. Not all retained metallic fragments require extraction: ballistic literature supports selective retention of asymptomatic fragments when removal would cause greater morbidity, with removal reserved for particular locations or complications [5]. Computed tomography provides the best anatomical definition of a radiopaque foreign body and associated injury, while serial plain radiographs can demonstrate interval migration but provide limited depth information [3, 6].

For haemodynamically stable patients without generalized peritonitis, laparoscopy provides both diagnosis and treatment while allowing systematic inspection of the abdominal cavity. Compared with laparotomy in selected abdominal trauma patients, meta-analysis has associated laparoscopy with fewer wound infections and a shorter hospital stay, by approximately 3.8 days, although study heterogeneity is substantial [7]. Laparotomy remains appropriate for haemodynamic instability, generalized peritonitis, suspected major vascular injury, or when exposure, anatomy or operative progress is inadequate for safe minimally invasive management.

The principal laparoscopic pitfall is missed injury, particularly hollow-viscus or mesenteric injury. An older prospective study identified only 57% of significant injuries at diagnostic laparoscopy and reported sensitivity below 50% for hollow-viscus injury [8]; contemporary pooled data suggest a much lower overall missed-injury rate of approximately 0.5% in appropriately selected trauma patients [7]. Surgeons should therefore perform a deliberate bowel and mesenteric survey, be prepared to repair bowel or vascular injury laparoscopically, and convert promptly when safe assessment or treatment cannot be achieved. In the present case, targeted intraoperative radiography with a laparoscopic instrument as a radiopaque reference enabled accurate localization when direct inspection failed, supporting its use as an accessible adjunct for small metallic foreign bodies [6, 9, 10].

## Supplementary Material

Video_1_video_of_removal_of_needle_rjag1012
